# Vitamin D Improves Nitric Oxide-Dependent Vasodilation in Adipose Tissue Arterioles from Bariatric Surgery Patients

**DOI:** 10.3390/nu11102521

**Published:** 2019-10-18

**Authors:** Abeer M. Mahmoud, Mary Szczurek, Chandra Hassan, Mario Masrur, Antonio Gangemi, Shane A. Phillips

**Affiliations:** 1 Division of Endocrinology, Diabetes and Metabolism, Department of Medicine, University of Illinois at Chicago, Chicago, IL 60612, USA; shanep@uic.edu; 2 Department of Physical Therapy, University of Illinois at Chicago, Chicago, IL 60612, USA; mszczurek@uic.edu; 3 Integrative Physiology Laboratory, College of Applied Health Sciences, University of Illinois at Chicago, Chicago, IL 60612, USA; 4 Department of Surgery, University of Illinois at Chicago, Chicago, IL 60612, USA; chandrar@uic.edu (C.H.); mmasrur@uic.edu (M.M.); gangemi@uic.edu (A.G.)

**Keywords:** vitamin D, obesity, microvascular, bariatric surgery, weight loss, nitric oxide

## Abstract

There is a high prevalence of vitamin-D deficiency in obese individuals that could be attributed to vitamin-D sequestration in the adipose tissue. Associations between vitamin-D deficiency and unfavorable cardiometabolic outcomes were reported. However, the pathophysiological mechanisms behind these associations are yet to be established. In our previous studies, we demonstrated microvascular dysfunction in obese adults that was associated with reduced nitric oxide (NO) production. Herein, we examined the role of vitamin D in mitigating microvascular function in morbidly obese adults before and after weight loss surgery. We obtained subcutaneous (SAT) and visceral adipose tissue (VAT) biopsies from bariatric patients at the time of surgery (*n* = 15) and gluteal SAT samples three months post-surgery (*n* = 8). Flow-induced dilation (FID) and acetylcholine-induced dilation (AChID) and NO production were measured in the AT-isolated arterioles ± NO synthase inhibitor N(ω)-nitro-L-arginine methyl ester (L-NAME), hydrogen peroxide (H_2_O_2_) inhibitor, polyethylene glycol-modified catalase (PEG-CAT), or 1,25-dihydroxyvitamin D. Vitamin D improved FID, AChID, and NO production in AT-isolated arterioles at time of surgery; these effects were abolished by L-NAME but not by PEG-CAT. Vitamin-D-mediated improvements were of a higher magnitude in VAT compared to SAT arterioles. After surgery, significant improvements in FID, AChID, NO production, and NO sensitivity were observed. Vitamin-D-induced changes were of a lower magnitude compared to those from the time of surgery. In conclusion, vitamin D improved NO-dependent arteriolar vasodilation in obese adults; this effect was more significant before surgery-induced weight loss.

## 1. Introduction

Obesity and vitamin-D deficiency are two major global public health concerns that affect over one-third of the population. A body mass index of more than 30 kg/m^2^ is associated with lower serum vitamin-D levels compared with non-obese individuals [1]. Several mechanisms that contribute to the high incidence of vitamin-D deficiency in obese people were suggested. Ultraviolet radiation from sunlight exposure is required for cutaneous production of vitamin D [2]. It was proposed that obese individuals, overall, have suboptimal levels of vitamin D because they tend to participate in fewer outdoor activities and, accordingly, have less exposure to sunlight. Vitamin D is a lipid-soluble vitamin, and it is thought that the lower bioavailability of vitamin D in obese individuals, compared to lean individuals, is due to vitamin-D sequestration within the adipose tissues [3]. Lastly, vitamin-D deficiency may also be due to inadequate dietary intake or malabsorption of vitamin D [4,5]. Obesity is a well-established risk factor for the development of cardiovascular disease (CVD), and several mechanisms may contribute to this link. However, a role of vitamin-D deficiency in obesity-associated cardiovascular (CV) risk is yet to be established. 

Epidemiological data reported associations between vitamin D deficiency and left ventricular hypertrophy, hypertension, increased arterial stiffness, and endothelial dysfunction [4,6,7,8,9,10,11,12]. The pathophysiological underpinnings of these associations remain mostly unexplained. The most reliable and convincing evidence for the involvement of vitamin D in the pathogenesis of CVD comes from studies demonstrating an association between vitamin-D deficiency and hypertension [13]. Vitamin-D deficiency is associated with a higher risk for preeclampsia, a condition that is characterized by hypertension [14]. Also, a significant inverse association was reported between vitamin-D serum levels and arterial blood pressure in the elderly [15]. An interventional study demonstrated that one month of vitamin-D intake (1,5000 IU/day) reduced tissue sensitivity to the stimulation of the renin–angiotensin system manifested by improved renal blood flow and reduced mean arterial pressure during an infusion of angiotensin II [16]. Moreover, vitamin-D intake (2000 IU/day) for 14 days in healthy adults reduced augmentation index, a measure of arterial stiffness that contributes to the development of hypertension [17].

The discovery of vitamin-D receptor (VDR) widened the scope of biological effects that vitamin D plays in human health [18]. VDR is a transcription factor that enters the nucleus upon binding to vitamin D, where it attaches to specific DNA regions and activates the transcription of a myriad of genes that coordinate several biological responses [18]. VDR is widely expressed throughout the human body in almost all cells and tissues, including vascular smooth muscle cells, cardiomyocytes, and endothelial cells [19]. Animal studies found that loss of VDR signaling increased arterial stiffness and elevated systolic and diastolic blood pressure, independent of the renin–angiotensin–aldosterone system [20]. Another study found that VDR knockout mice have increased arterial stiffness through decreased bioavailability of nitric oxide (NO) [21]. These and other studies proposed that the leading mechanism for the association between vitamin-D deficiency and CVD is related to endothelial dysfunction and impaired endothelial-dependent NO production [22,23,24]. 

Despite this accumulating evidence of the role of vitamin D in vascular function, direct mechanistic evidence of this role in the microvasculature of morbidly obese population and how it might be modified following massive weight loss are largely unexplored. The purpose of the current study is to investigate the effects of vitamin D on the microvasculature isolated from both subcutaneous (SAT) and visceral adipose tissue (VAT) in morbidly obese adults before and after bariatric surgery-induced weight loss. The central hypothesis is that vitamin D would improve microvascular function in obese adults via improving flow-mediated NO production, and this effect would be of a greater magnitude before compared to after weight loss. In our previous work, we found that flow-induced dilation (FID) is impaired in morbidly obese individuals due to an imbalance of NO and H_2_O_2_ [25,26]. H_2_O_2_ may serve as a compensatory mechanism for vasodilation during pathological conditions such as obesity [27]. Using the proposed ex vivo system in this study, we are able to explore the contributing vasoactive mediators of vitamin-D effects on microvascular function, mainly NO and H_2_O_2_. 

## 2. Methods

### 2.1. Human Participants

**Figure 1:**
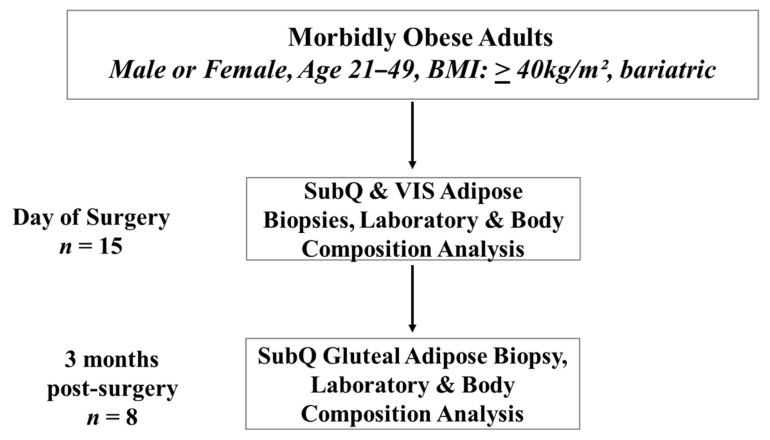
Study flow chart.

Subjects were 15 men (*n* = 2) and women (*n* = 13), who underwent laparoscopic bariatric surgery at the University of Illinois Medical Center. The age of subjects ranged from 21 to 49 years old, and all the women were premenopausal. The eligibility criteria included a body mass index (BMI) of at least 40 kg/m^2^ and the absence of significant chronic morbidities or inflammatory conditions that may have confounding effects on the study outcomes. Excluded subjects included pregnant women and individuals with current diabetes mellitus (type I or II), heart disease, liver disease, kidney disease, gallbladder disease, cancer, or acute or chronic inflammatory diseases such as rheumatoid arthritis. Four subjects were taking vitamin D supplementation (10,000–50,000 IU vitamin D/week). Determination of the subject’s eligibility criteria was completed before the pre-surgery evaluation clinical visit. At the clinical visit, eligible subjects were informed about the study details, and those who were interested in participating provided written informed consent. The study protocol and procedures were approved by the University of Illinois at Chicago Institutional Review Board and followed the standards set by the latest revision of the Declaration of Helsinki. A post-surgery visit at the University of Illinois Clinical Interface Core (UIC CIC) was established with each subject during the time of written consent. The study flowchart detailing subject numbers, the timeline for biopsy, and clinical data collection is displayed in Figure 1.

### 2.2. Physical and Cardiometabolic Measurements

Physical characteristics, including age, gender, body weight, height, BMI, waist circumference, and cardiometabolic risk factors, were assessed. Fasting blood samples were obtained before biopsy acquisition for measuring biochemical parameters (lipid profile and glucose metabolism).

### 2.3. Sample Acquisition

On the day of bariatric surgery, blood samples were collected before the administration of anesthesia. After the administration of anesthesia, adipose tissue samples (SAT and VAT) were collected by the surgeon. All samples were immediately placed in cold 2-(4-(2-hydroxyethyl) piperazin-1-yl) ethanesulfonic acid (HEPES) buffer solution to maintain the viability of the tissue. In the post-surgery visit, all subjects fasted for 12 hours before the study visit. At the visit, subjects’ anthropometric measurements, blood pressure, and heart rate were measured. A fasting blood draw was obtained for total cholesterol, triglycerides, glucose, and insulin measurements. During the post-surgery visit, a SAT biopsy was obtained by a certified nurse practitioner from the gluteal region under local anesthesia. The biopsy was immediately placed in cold HEPES buffer solution. 

### 2.4. Microvascular Preparation

Adipose tissues were dissected, and resistance arterioles were isolated and cleaned of excess fat and connective tissues. Arterioles were then washed and prepared for measuring changes in the internal diameter in response to flow and acetylcholine (Ach) as previously described [26,28,29]. In summary, isolated arterioles were cannulated using glass micropipettes in an organ perfusion chamber, and both ends were secured using a 10-0 nylon Ethilon monofilament suture. Cannulated arterioles within the organ chamber were then placed on the stage of an inverted microscope attached to a video camera, a video monitor, and a video measuring device (model VIA-100; Boeckeler, Madison, WI, USA). The organ chamber was perfused with heated physiological salt solution (Krebs buffer) that contained the following components (mM): 4.4 KCL, 123 NaCl, 2.5 CaCl_2_, 20 NaHCO_3_, 1.2 MgSO_4_, 1.2 KH_2_PO_4_, and 11 glucose. The pH of the solution was kept at 7.4 ± 0.05, and the temperature was maintained at 37 °C. The buffer was also supplied with air mixture of 21% O_2_, 5% CO_2_, and 74% N_2_. Each end of the cannulated arteriole was connected via silicon tubes to a physiological buffer-containing reservoir, and the intraluminal pressure gradient (10–100 cm H_2_O) was established by modifying the distance between both reservoirs in equal and opposite directions [30]. 

### 2.5. Measurements of Flow-Induced Dilation

Arterioles were constricted with endothelin-1 (Peninsula, San Carlos, CA, USA), and those constricted less than 30% were excluded from the study. The internal diameter of the cannulated arterioles was measured at baseline and during gradual increases of the intraluminal pressure gradient (10–100 cm H_2_O) or acetylcholine concentration (ACh; 10^−9^ to 10^−4^ M) [26]. Measurements were repeated after incubations with 1,25-dihydroxyvitamin D (1-25(OH)_2_D; vitamin D, 1 nM), the eNOS (endothelial nitric oxide synthase) inhibitor L-NAME (10^−4^ M), the H_2_O_2_ scavenger PEG-CAT (500U/mL), L-NAME plus vitamin D, or PEG-CAT plus vitamin D. Treatments were added to the physiological bathing solution in 30 minutes before FID and acetylcholine-induced dilation (AchID) measurements were obtained. Papaverine (10^−4^ M) was used at the end of each experiment to measure maximal vasodilation. We reported percentage vasodilation as the percentage increase in the arteriolar diameter after each treatment condition relative to the endothelin-1 (ET-1)-constricted state.

### 2.6. Measurements of Arteriolar NO

Nitric oxide produced by freshly isolated microvessels was measured as previously described [31] using Enzo Life Sciences NO Detection Kit (ThermoFisher Scientific, MA, USA). NO measurements were performed in cannulated vessels in the organ chambers. In order to stimulate NO production, vessels were exposed to a pressure gradient of Δ60 cm H_2_O during which they were incubated with the NO detection reagents. This step was followed by vessel excision, washing, and mounting on microscopic coverslips. Images were taken immediately using fluorescence microscopy (Eclipse TE 2000, Nikon, Japan) at 650/670 nm. All incubations and staining and detection protocols were fixed in all experiments. The acquired images were then analyzed for the intensity of the fluorescent signal in arbitrary units using National Institute of Health (NIH) Image J software (NIH, Bethesda, MD, USA).

### 2.7. Statistical Analyses

All results are reported as means ± standard error, and *p* < 0.05 was considered statistically significant. Fluorescent images were analyzed for fluorescence intensity after correcting for background autofluorescence using NIH Image J software (NIH, Bethesda, MD, USA). Paired measurements for FID, AChID, NO fluorescence, physical characteristics, and cardiometabolic parameters were assessed using Student’s paired *t*-test for within-group comparisons. One-way ANOVA followed by an appropriate post hoc test was used when there were more than two comparisons among different vessel treatments. Dilation in dose–response data was presented as a percentage increase in the arteriolar diameter after each treatment condition relative to the ET-1-constricted state. Analyses were performed using SPSS statistical package (version 18.0; SPSS Inc, Chicago, IL, USA). 

## 3. Results

### 3.1. Physical and Cardiometabolic Parameters

Table 1 summarizes age, gender, and anthropometric characteristics of all participants. Fifteen subjects (13 females and two males; age: 37 ± 8 years) were enrolled in the study. Eight subjects (seven female and one male; age: 35 ± 6 years) participated in the post-surgery visit. Study participants lost 13.3 kg of their body weight on average, and their BMI and waist circumference decreased by 13.6% and 12%, respectively. Table 2 summarizes the cardiometabolic parameters we measured in the study, including blood pressure, heart rate, lipid profile, and glucose metabolism. After surgery, both systolic and diastolic blood pressures decreased significantly. On average, total cholesterol and glucose decreased by ~10% and 4%, respectively, three months after surgery compared to the day of surgery. Serum levels of vitamin D increased significantly (31.8%, *p* = 0.0003) after bariatric surgery.

### 3.2. Effect of Vitamin D on Arteriolar FID, AchID, and NO Production before Weight Loss (at Time of Surgery)

**Figure 2:**
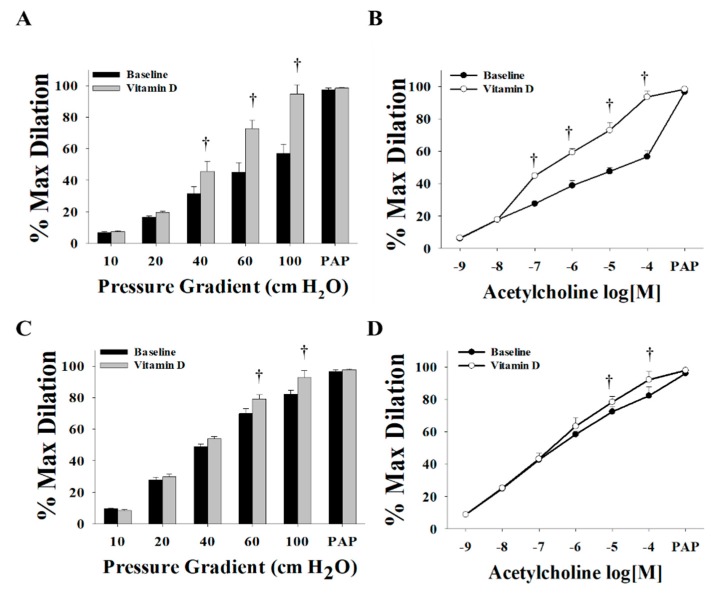
Effect of vitamin D on flow-induced dilation (FID) and acetylcholine-induced dilation (AChID) in adipose tissue resistance arterioles collected on the day of surgery (*n* = 15 subjects). FID measurements in visceral adipose tissue (VAT) arterioles (**A**) and subcutaneous adipose tissue (SAT) arterioles (**C**) corresponding to increasing intraluminal pressure gradients of 10–100 cm H_2_O. AchID measurements in VAT arterioles (**B**) and SAT arterioles (**D**) corresponding to increasing concentrations of acetylcholine (Ach) (10^−9^ to 10^−4^ M). All measurements are presented as means ± standard error (SE); † *p* < 0.05 comparing vitamin D with baseline.

**Figure 3:**
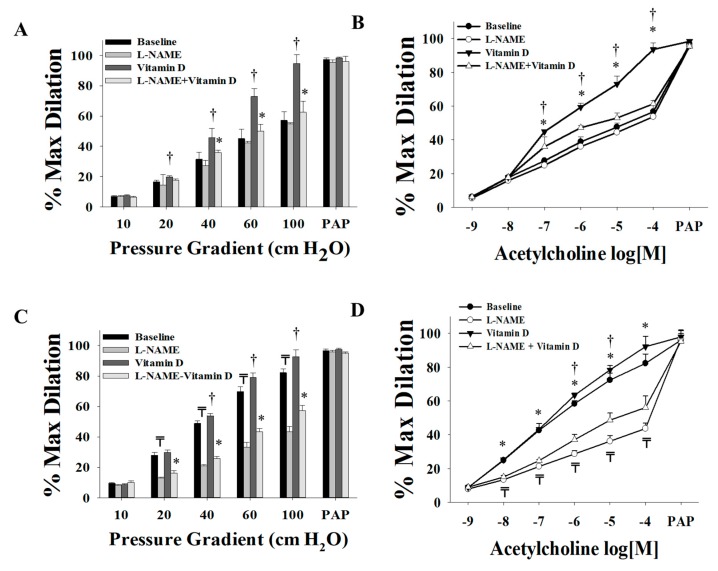
Effect of endothelial nitric oxide synthase (eNOS) inhibition via L-NAME on FID and AchID in adipose tissue resistance arterioles collected on the day of surgery (*n* = 15 subjects). FID measurements corresponding to intraluminal pressure gradients of 10–100 cm H_2_O in VAT arterioles (**A**) and SAT arterioles (**C**) with and without vitamin D incubation. AchID measurements corresponding to increasing Ach concentrations (10^−9^ to 10^−4^ M) in VAT arterioles (**B**) and SAT arterioles (**D**) with and without vitamin-D incubation. All measurements are presented as means ± standard error (SE); ₸ *p* < 0.05 comparing L-NAME with baseline, † *p* < 0.05 comparing vitamin D with baseline, and * *p* < 0.05 comparing L-NAME + vitamin D with vitamin D alone.

Vitamin D enhanced the FID and AChID measurements in arterioles (*n* = 15) isolated from both VAT (Figure 2A,B) and SAT (Figure 2C,D) compared to baseline. These improvements were of a higher magnitude in the VAT compared to SAT arterioles. At ∆60 cm H_2_O, the average FID increased by 60% in VAT arterioles and 14% in SAT arterioles. Similar results were obtained in response to Ach. Also, vasodilation improvements in VAT arterioles were obtained at lower pressure gradient (∆40) compared to SAT arterioles where significant improvements were only achieved at ∆60 and higher. These findings could be explained by the lower baseline FID and AchID measurements in VAT compared to SAT arterioles. These impaired measurements provide a chance for a more perceptible magnitude of improvement in the VAT arterioles in response to vitamin D. Previous data from our lab [32] recapitulated the same phenomenon of impaired dilation responses of VAT arterioles in response to flow and acetylcholine compared to SAT arterioles. Moreover, unlike the significant reduction in FID and AChID observed in SAT arterioles in response to endothelial nitric oxide synthase (eNOS) inhibition by L-NAME (Figure 3C,D), no significant response was observed in VAT arterioles (Figure 3A,B). The observed low sensitivity of VAT arterioles to NO inhibition might indicate a disruption in the NO-dependent vasodilation mechanism in these arterioles. 

Our data show an enhanced NO sensitivity in VAT arterioles in response to vitamin D as evident by significant reductions in FID and AchID in vitamin-D-treated VAT arterioles in response to L-NAME (Figure 3A,B). Vitamin-D-induced FID and AChID improvements were also abolished in SAT arterioles in response to L-NAME. Unlike VAT arterioles, the magnitude of L-NAME-induced inhibition in vitamin-D-treated SAT arterioles is comparable to that of the untreated SAT arterioles, indicating a lesser effect of vitamin D on improving NO pathway in SAT arterioles (Figure 3A–D). 

**Figure 4:**
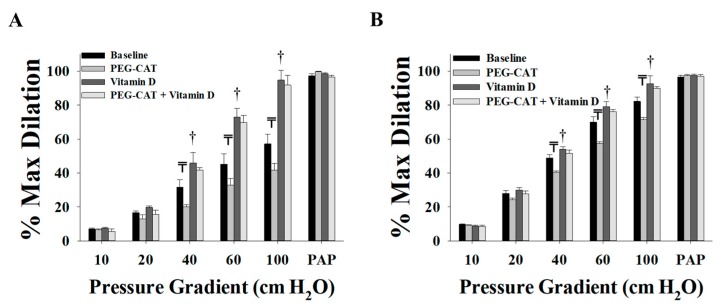
Effect of scavenging H_2_O_2_ via PEG-CAT on FID in adipose tissue resistance arterioles collected on the day of surgery (*n* = 15). FID measurements corresponding to intraluminal pressure gradients of 10–100 cm H_2_O in VAT arterioles (**A**) and SAT arterioles (**B**) with and without vitamin-D incubation. All measurements are presented as means ± standard error (SE); ₸ *p* < 0.05 comparing L-NAME with baseline, and † *p* < 0.05 comparing vitamin D with baseline.

**Figure 5:**
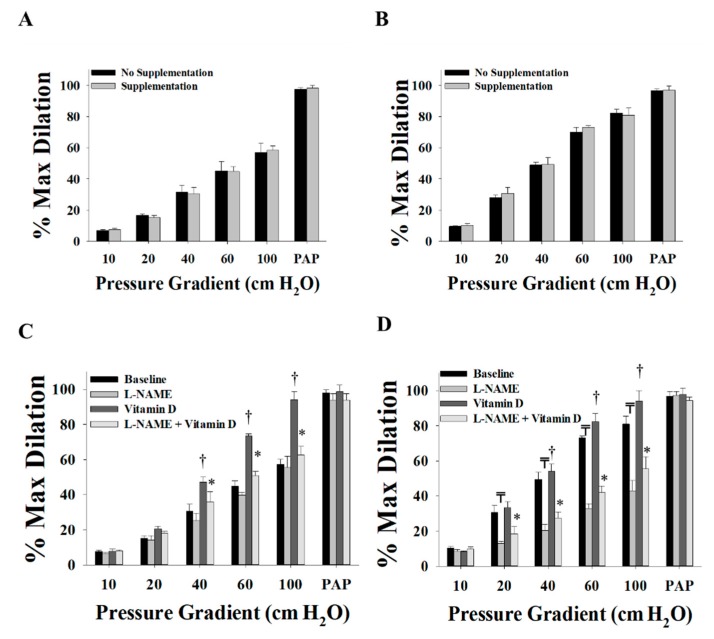
Effect of vitamin-D supplementation prior to surgery on FID in adipose tissue resistance arterioles collected on the day of surgery. FID measurements corresponding to intraluminal pressure gradients of 10–100 cm H_2_O in VAT arterioles (**A**) and SAT arterioles (**B**) from subjects who administered vitamin-D supplements before surgery (*n* = 4) and subjects who did not (*n* = 11). Effect of L-NAME and vitamin-D incubation on FID in VAT arterioles (**C**) and SAT arterioles (**D**) from subjects who administered vitamin-D supplements before surgery (*n* = 4). All measurements are presented as means ± standard error (SE); ₸ *p* < 0.05 comparing L-NAME with baseline, † *p* < 0.05 comparing vitamin D with baseline, and * *p* < 0.05 comparing L-NAME + vitamin D with vitamin D alone.

It was established that H_2_O_2_ is a vasoactive mediator that is released from the endothelium as a compensatory mechanism for impaired NO-mediated vasodilation [27,33,34]. This compensatory mechanism mediates vasodilation, at least partly, in conditions characterized by inflammation and increased production of reactive oxygen species (ROS) such as obesity, hypertension, and coronary artery disease [26,31]. Our data show that scavenging H_2_O_2_ via PEG-CAT decreased FID significantly in both VAT (Figure 4A) and SAT (Figure 4B) arterioles, indicating a possible contribution of H_2_O_2_ in the arteriolar FID in the obese subjects of this study. On the other hand, vitamin-D-treated vessels lost the response to the H_2_O_2_ scavenger, PEG-CAT (Figure 4A,B), which might refer to an effect of vitamin D in reducing ROS production or arteriolar dependence on H_2_O_2_ as a vasodilator. In the four subjects who were administered prescribed vitamin-D supplementation before surgery (10000 IU, *n* = 1; 5,0000 IU, *n* = 3), we observed no differences in arteriolar FID compared to those who were not taking vitamin-D supplementation (Figure 5A,B). Also, the effect of L-NAME on inhibiting vasodilation in participants taking vitamin-D supplementation (Figure 5C,D) followed the same patterns observed in all participants (Figure 3). 

**Figure 6:**
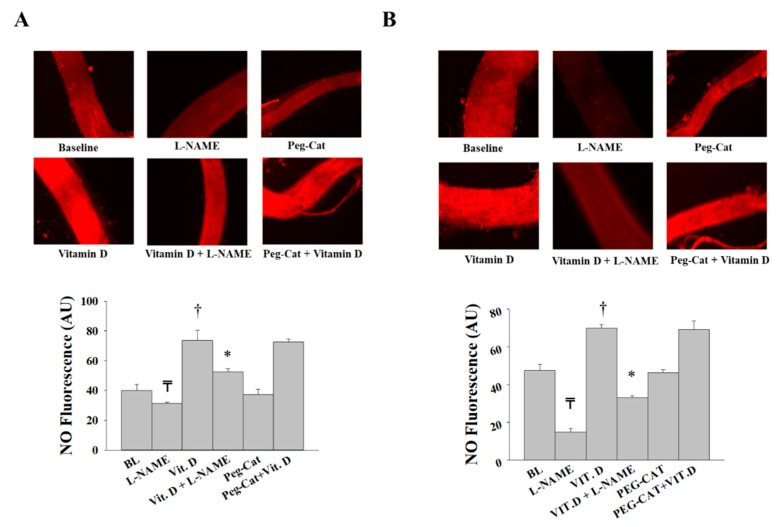
Nitric oxide (NO) production in isolated adipose tissue arterioles collected on the day of surgery. Representative images by fluorescence microscopy of NO generation conditions at baseline and after incubation with L-NAME, PEG-CAT, vitamin D, vitamin D plus L-NAME, and vitamin D plus PEG-CAT in VAT arterioles (**A**) and SAT arterioles (**B**). Charts represent NO fluorescent signals that were measured and expressed in arbitrary units using NIH Image J software. All measures are represented as means ± SE; ₸ *p* < 0.05 comparing L-NAME with baseline, † *p* < 0.05 comparing vitamin D with baseline, * *p* < 0.05 comparing L-NAME + vitamin D with vitamin D alone.

Consistent with the FID and AchID data, NO production was attenuated in response to L-NAME incubation, albeit to a greater extent in SAT (−64%) compared to VAT (−20%) arterioles. In the presence of vitamin D, the production of NO increased in both VAT and SAT arterioles (Figure 6A,B, respectively). This increase was of a higher magnitude in VAT (75%) than SAT (50%) arterioles, which follows the pattern of FID changes we presented above. These increases in NO production were abolished by L-NAME, albeit to a greater extent in SAT (−57%) compared to VAT (−29%) arterioles. PEG-CAT did not induce any significant effects on NO production in either SAT or VAT arterioles. In summary, vasodilation in SAT arterioles is more dependent on NO and more sensitive to NO inhibition than VAT arterioles, and the average baseline NO production in VAT arterioles was lower than SAT arterioles. Therefore, by improving NO production, vitamin D enhanced vasodilation of VAT arterioles to a greater extent than SAT arterioles and increased their sensitivity to NO inhibition. 

### 3.3. Effect of Vitamin D on Arteriolar FID, AchID, and NO Production after Weight Loss (Three Months after Surgery)

**Figure 7:**
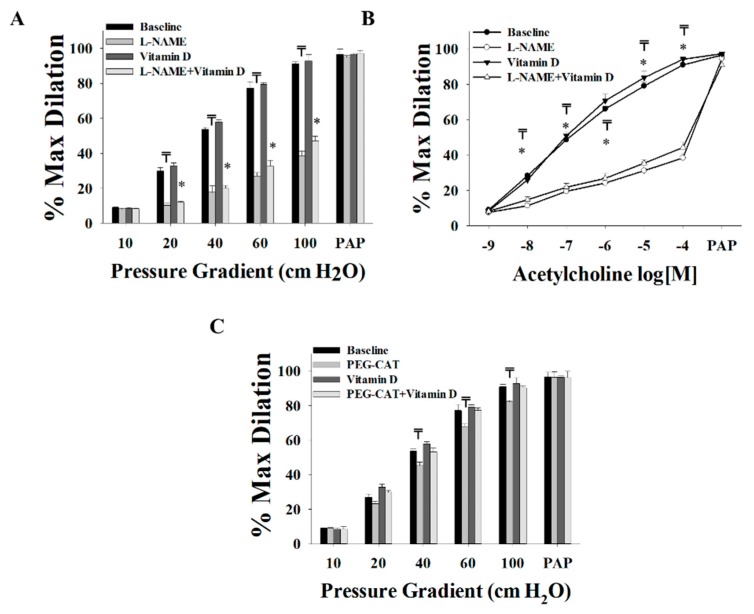
Effect of vitamin D, L-NAME, and PEG-CAT on vasodilation of SAT arterioles collected three months after bariatric surgery (*n* = 8). FID measurements corresponding to intraluminal pressure gradients of 10–100 cm H_2_O in SAT arterioles at baseline and after incubation with L-NAME, PEG-CAT, vitamin D, vitamin D plus L-NAME, and vitamin D plus PEG-CAT (**A**,**C**). AchID measurements corresponding to increasing Ach concentrations (10^−9^ to 10^−4^ M) in SAT arterioles at baseline and after incubation with L-NAME, vitamin D, and vitamin D plus L-NAME (**B**). All measures are represented as means ± SE; ₸ *p* < 0.05 comparing L-NAME or PEG-CAT with baseline, and * *p* < 0.05 comparing L-NAME + vitamin D with vitamin D alone.

Post-surgery SAT arterioles showed minimal to no improvements in FID (Figure 7A) or AChID (Figure 7B) in response to vitamin D. Baseline arteriolar dilation was attenuated in response to NO inhibition via L-NAME, to a higher degree (−67% in ∆60) than that in arterioles obtained during surgery (−52% in ∆60). These findings might indicate a role of surgical weight loss in improving NO generation in SAT arterioles. The H_2_O_2_ scavenger, PEG-CAT, reduced the FID in post-surgery SAT arterioles to a lesser extent (−9% at Δ60, Figure 7C) than those obtained at the time of surgery (−20% at Δ60, Figure 4B), indicating less dependence on H_2_O_2_ as a vasodilator after surgical weight loss. This effect was further enhanced by vitamin-D incubation as evident by a complete loss of response to PEG-CAT in these arterioles (Figure 7C). 

**Figure 8:**
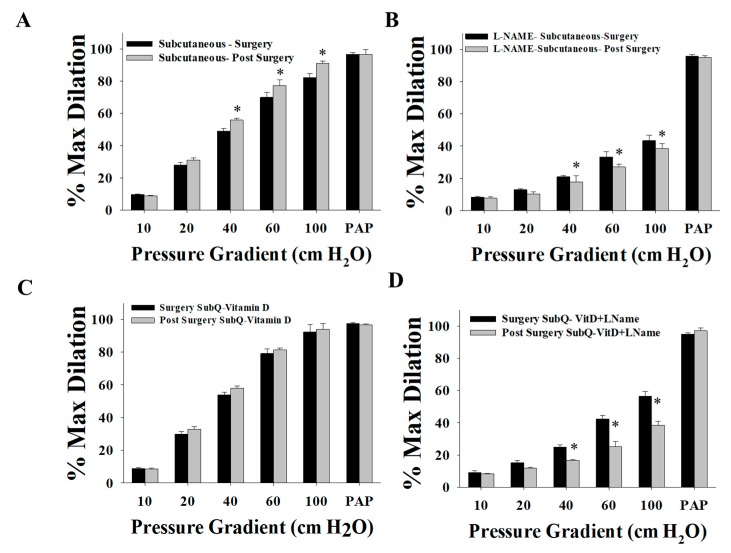
Comparison of the FID between SAT arterioles collected on the day of bariatric surgery and those collected three months after surgery. FID measurements corresponding to intraluminal pressure gradients of 10–100 cm H_2_O in SAT arterioles at baseline (**A**) and after incubation with L-NAME (**B**), vitamin D (**C**), and vitamin D plus L-NAME (**D**). All measures are represented as means ± SE; * *p* < 0.05 comparing post-surgery with surgery-obtained arterioles.

**Figure 9:**
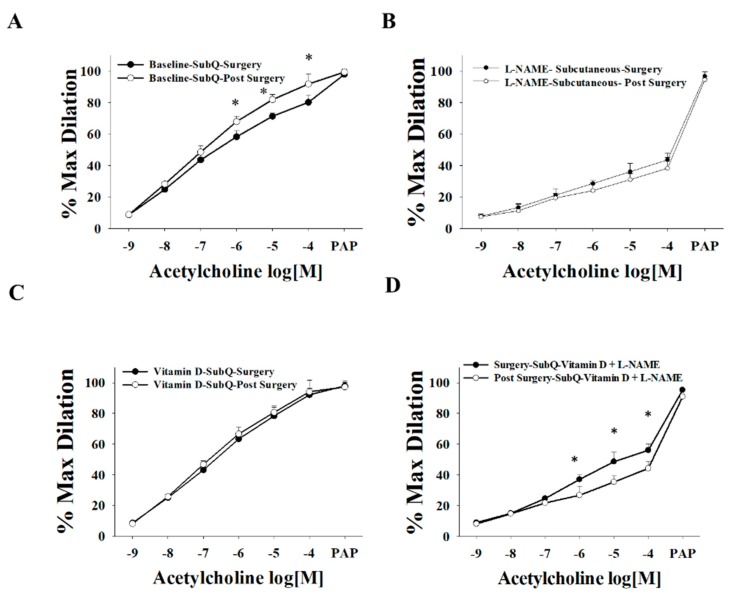
Comparison of the AchID between SAT arterioles collected on the day of bariatric surgery and those collected three months after surgery. AchID measurements corresponding to increasing Ach concentrations (10^−9^ to 10^−4^ M) in SAT arterioles at baseline (**A**) and after incubation with L-NAME (**B**), vitamin D (**C**), and vitamin D plus L-NAME (**D**). All measures are represented as means ± SE; * *p* < 0.05 comparing post-surgery with surgery-obtained arterioles.

When compared to those collected on the day of surgery, post-surgery SAT arterioles demonstrated improved FID (Figure 8A) at baseline and an enhanced response to the eNOS inhibitor, L-NAME (Figure 8B). This effect can also be observed when comparing the percentage of FID reduction after L-NAME in the post-surgery arterioles (Δ60, −68%, Figure 7A) to that in the surgery arterioles (Δ60, −57%, Figure 3C). After incubation with vitamin D, FID in surgery and post-surgery SAT arterioles was comparable even though FID in post-surgery SAT arterioles did not improve in response to vitamin D (Figure 8C). These results may indicate that the effect of vitamin D on improving FID before weight loss is comparable to the effect of weight loss three months after bariatric surgery. Furthermore, additional augmentation by vitamin D could not be achieved following weight loss surgery. Enhanced sensitivity of SAT arterioles to L-NAME was observed in response to a combined effect of weight loss and vitamin D compared to the sole effect of vitamin D (Figure 8D). In post-surgery arterioles, the response to Ach mirrored that of FID (Figure 9).

**Figure 10:**
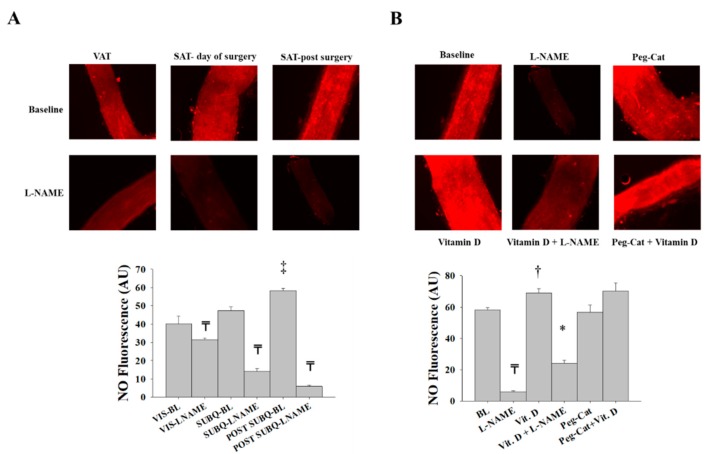
NO production in isolated adipose tissue arterioles. (**A**) Representative images by fluorescence microscopy of NO generation conditions at baseline and after incubation with L-NAME in adipose tissue arterioles collected on the day of surgery (VAT and SAT day of surgery) and three months after surgery (SAT post-surgery). (**B**) Representative fluorescence microscopy images of NO generation conditions at baseline and after incubation with L-NAME, PEG-CAT, vitamin D, vitamin D plus L-NAME, and vitamin D plus PEG-CAT in SAT arterioles collected three months after surgery. Charts represent NO fluorescent signals that were measured and expressed in arbitrary units using NIH Image J software. All measures are represented as means ± SE; ₸ *p* < 0.05 comparing L-NAME with corresponding baseline in VAT, SAT day of surgery, and SAT post-surgery, ‡ *p* < 0.05 comparing SAT day of surgery and SAT post-surgery, † *p* < 0.05 comparing vitamin D with baseline, and * *p* < 0.05 comparing L-NAME + vitamin D with vitamin D alone.

Nitric oxide fluorescence was higher in SAT arterioles obtained post-surgery compared to arterioles obtained during surgery, and a more significant reduction in NO production in response to L-NAME was observed in post-surgery SAT arterioles (Figure 10A). Similar to FID data, NO production was comparable in SAT arterioles obtained during and after surgery and incubated with vitamin D (Figure 6A and Figure 10B). In the presence of a combined weight loss and vitamin D, a greater reduction in NO production in response to L-NAME was observed in SAT arterioles from post-surgery (−69%) vs. surgery (−54%) (Figure 6B and Figure 10B). When arterioles from surgery samples were incubated with vitamin D, there was an enhancement in NO production (40%); however, after weight loss surgery, vitamin-D-induced improvements in NO production were significantly lower (15%). In summary, both NO and H_2_O_2_ play a role in vasodilation in SAT arterioles at the time of surgery in obese bariatric patients. After surgical weight loss, the NO vasodilatory component is enhanced, and the H_2_O_2_ compensatory component is reduced. Vitamin D has a more prominent effect in improving vasodilation and NO production and sensitivity in SAT arterioles obtained before weight loss compared to those collected after weight loss. 

## 4. Discussion

The main findings of the current study are that vitamin D improved FID and AChID and increased NO production in resistance arterioles from SAT and VAT. These effects were abolished by eNOS inhibition via L-NAME but not by H_2_O_2_ scavenging via PEG-CAT. In comparing the measures from pre- and post-surgery, the primary findings of the study are that (1) subjects lost a significant percentage of their body weight three months after bariatric surgery, (2) SAT arterioles from post-surgery biopsies demonstrated improved FID and AChID at baseline, increased NO production, enhanced sensitivity to L-NAME, and reduced effect of PEG-CAT compared to SAT arterioles from the day of surgery sample, (3) in post-surgery SAT arterioles, vitamin D did not induce any further enhancements in vasodilation or NO production, and (4) a synergistic interaction between weight loss and vitamin-D incubation was observed in relation to arteriolar sensitivity to eNOS inhibition via L-NAME.

The findings that vitamin D improves FID, AChID, and NO fluorescence in SAT and VAT support the notion that vitamin D is a regulator of endothelial function [35,36]. Our findings are consistent with previous studies suggesting that vitamin D is a transcriptional regulator of eNOS, effectively increasing the production of NO, the most potent vasodilator within the vasculature [37,38]. Previous studies showed that vitamin-D receptors play a critical role in maintaining vascular health, and this assumption was supported by data showing reduced NO production, endothelial dysfunction, increased arterial stiffness, increased aortic impedance, and structural remodeling of the aorta in mice with mutant vitamin-D receptors [21]. Also, earlier studies demonstrated hypertension and myocardial hypertrophy in vitamin-D receptor knockout mice [39]. In vitro experiments conducted by Martínez-Miguel et al. [40] also supported the role of vitamin-D supplementation in inducing eNOS transcription and activity in endothelial cells. In addition to the effect of vitamin D on inducing eNOS expression, it was suggested that vitamin D improves vascular function via reducing the production of NO-scavenging oxygen radicals and subsequently improving NO bioavailability [41,42,43]. 

In addition to its role in inducing eNOS expression and activity, vitamin D was shown to reduce nuclear factor kappa B (NF-κB) activity and inhibit the production of several pro-inflammatory cytokines such as tumor necrosis factor-α (TNF-α), interferon gamma (IFN-γ), interleukin 1 beta (IL-1β), and IL-8, while upregulating the anti-inflammatory cytokine, IL-10 [44]. Previous studies showed that vitamin D inhibits cytokine-mediated endothelial cell activation and the production of surface adhesion molecules [45]. Furthermore, vitamin D’s protective effect against atherosclerosis and hypertension was explained by its ability to decrease the proliferation of vascular smooth muscle, decrease calcium influx into endothelial cells, decrease vascular resistance, and regulate the renin–angiotensin system [46,47].

In addition to the experimental evidence, several epidemiological studies linked low vitamin-D levels with high blood pressure, coronary artery disease, myocardial infarction, and heart failure [48,49,50]. Despite this experimental and epidemiological evidence, results from clinical trials investigating the effect of vitamin-D supplementation on improving vascular function and reducing cardiovascular risk were less encouraging. In a metanalysis by Beveridge et al. [51], effects of vitamin-D supplementation on flow-mediated dilatation of the brachial artery, pulse wave velocity, augmentation index, central blood pressure, microvascular function, and reactive hyperemia index were assessed using data from 31 trials (2751 participants). This metanalysis showed no significant improvements in macrovascular function and modest improvements in microvascular function in response to vitamin-D administration in a daily dose that ranged from 900 to 5000 IU for a duration of four weeks to 12 months. Other metanalyses showed a similar lack of significant effects of vitamin-D supplementation on vascular function [52,53,54]. It is important to note that most of the trials included in these studies did not account for variations in biologic availability of the administered vitamin D that might be caused by different body compositions. It is possible that the lack of response to vitamin-D supplementation is due to low blood serum levels of vitamin D secondary to its sequestration within the adipose tissue. In support of this notion, previous studies reported lower serum bioavailability of vitamin-D supplementation in obese individuals [55,56]. Accordingly, it would be challenging to measure the systemic effects of vitamin D if it is sequestered in the body fat. In the current study, we sought to test the effect of direct exposure to vitamin D in endothelial function and the contributing vasoactive mediators in isolated human arterioles. Furthermore, we sought to test the effect of fat mass reduction after bariatric surgery on microvascular function and response to vitamin-D exposure. 

The demonstrated vascular improvements in response to exogenous (ex vivo) application of vitamin D on isolated blood vessels in the current study suggests that the lack of in vivo effects in previous clinical trials could be related to vitamin-D inaccessibility to vascular tissues. This supposition might also explain the observed lack of any significant differences in microvascular function between subjects who administered vitamin D before surgery and those who did not in the current study. Our findings also highlighted the mechanism via which vitamin D enhanced the microvascular function, which was found to be mainly mediated by a restoration of the production and sensitivity of the most potent endothelial-dependent vasodilator, NO. The effect of vitamin D on abolishing any dependence of microvascular dilation on H_2_O_2_, a compensatory mechanism for compromised NO pathways, indicates the role of vitamin D in reestablishing healthier vascular milieu and vasodilation mechanisms. The phenomenon of shifting the primary vasoactive mediator from NO to H_2_O_2_ under conditions characterized by inflammation and oxidative stress was reported by our research group and by others [26,27,31,57,58]. Despite being a vasodilator, H_2_O_2_ possesses proinflammatory, prothrombotic, and proatherogenic properties that eventually exacerbate vascular dysfunction [59]. Accordingly, the reduced sensitivity of arteriolar vasodilation to H_2_O_2_ scavenging might indicate a reduction in inflammation or ROS production or improved anti-oxidative mechanisms. However, future studies are required to confirm this assumption and explore the exact mechanism via which vitamin D reduces the dependence on H_2_O_2_ as a vasodilator. 

The findings that baseline FID, AChID, and NO fluorescence improved in SAT arterioles three months post-surgery and that no further improvements could be achieved in response to exogenous vitamin-D incubation suggest increased accessibility of vitamin D to vascular tissues after weight loss. This assumption is supported by the significant elevation of serum vitamin-D levels after surgery. However, this does not rule out other non-vitamin-D-related, weight loss-induced biological changes that might have contributed to the observed vascular improvements. Some of the previously suggested mechanisms could include an improved lipid profile and glucose metabolism and reduced systemic inflammation [60,61]. It is conceivable that the effect of weight loss in improving microvascular function is multifactorial; however, the results from the current study indicate a potential role of vitamin D in restoring vascular health after weight loss. We recently demonstrated the effect of dietary-induced weight loss in promoting microvascular health and endothelial-mediated NO production [29]. In the current study, we present evidence that vitamin D contributes, at least partially, to weight loss-associated vascular improvements. 

Findings from previous studies suggest that loss of visceral adipose tissue after weight loss interventions is easier and greater than subcutaneous adipose tissue [62,63] and that the latter is the primary storage site for cutaneously produced vitamin D [64]. In our study, weight loss three months post-surgery may not have affected the amount of subcutaneous adipose tissue significantly. Therefore, a significant amount of vitamin D sequestered in those tissues may not have been released into the serum. Accordingly, future studies with long-term follow-up are required to detect robust improvements in circulating vitamin D and vascular function. The response of FID to PEG-CAT decreased in the arterioles isolated after surgery compared to arterioles from the day of surgery. These findings are consistent with previous evidence indicating that in chronic diseases and morbid obesity, other endothelium-derived vasodilators, such as H_2_O_2_, may compensate for the lack of NO [26,27,34,65]. Yet, the current study is the first to demonstrate the effectiveness of surgical weight loss in shifting the major vasodilator back to NO and minimizing the dependence on H_2_O_2_ in human adipose tissue resistance arterioles. 

Although, consecutive to bariatric surgery, vitamin-D levels improved (Table 2), these levels are still considered less than adequate (>20 ng/mL) [66]. A growing body of evidence demonstrates a state of malabsorption and vitamin deficiencies following certain types of bariatric surgeries as reviewed by Lespessailles et al. [67]. For instance, bypassing the jejunum in the Roux-en-Y gastric bypass (RYGB) surgery and bile salt deficiency associated with bariatric surgery procedures affect vitamin-D absorption and bioavailability in post-bariatric patients [68,69]. Based on these facts, the 2008 interdisciplinary European guidelines on metabolic and bariatric surgery recommended the inclusion of vitamin D as a routine laboratory test that should be evaluated annually after bariatric surgery [70]. Also, the latest recommendations of the American Association of Clinical Endocrinologists, the Obesity Society, and American Society for Metabolic and Bariatric Surgery are to measure vitamin-D blood levels before and after bariatric surgery and to treat patients with 3000 IU of vitamin D daily after bariatric surgery to obtain a level greater than 30 ng/mL [71]. Obesity on one hand and post-bariatric malabsorption on the other hand might cause vitamin-D deficiency; therefore, future studies that investigate long-term vascular outcomes after bariatric surgery with and without vitamin-D supplementation are required.

There were several limitations to this study. Firstly, this study was limited to a young morbidly obese male and premenopausal female population, thereby limiting the generalizability of the findings to the population at large. Secondly, we had a relatively small sample size (pre-surgery: *n* = 15, post-surgery: *n* = 8), which carries with it the risk of a type II error due to low statistical power. Thirdly, the study design made it not possible to control for the menstrual cycle in the female subjects during sample acquisition. Hormonal changes during the menstrual cycle were shown to affect the macrovasculature, but its influence on the microcirculation is unknown. The surgery date could not be scheduled to control for this as a confounder a priori. Moreover, only eight of the 15 recruited subjects returned after their surgery for their second visit. No post-surgery VAT sample was obtained, as this biopsy was unattainable in the absence of general anesthesia. Another limitation of the current study is that pre- and post-surgery SAT samples were obtained from different adipose depots (abdominal vs. gluteal SAT). However, previous studies showed that mechanisms of dilations in SAT depots are not dependent on the region of biopsy [26]. In addition, the follow-up after surgery was for a short term (three months), which imposes difficulty in identifying long-term consequences of surgical weight loss. Thus, future investigations to evaluate the long-term vascular effects of weight loss after bariatric surgery are warranted. Finally, one of the main limitations of our study is the unbalanced female-to-male ratio. Although this study was not designed to determine gender- or racial/ethnic-specific differences in response to bariatric surgery, future studies are required to determine the influence of such variables on microvascular function.

In summary, this is the first study to explore the effects of surgical weight loss and vitamin D on microvascular function and the leading mechanisms of vasodilation in human isolated SAT and VAT resistance arterioles. The results of this study suggest a role of vitamin D in maintaining vascular health. It also suggests that weight loss is an integral component of enhancing vitamin-D bioavailability and its effects on enhancing microvascular function and, subsequently, the reduction of cardiovascular risk in morbidly obese individuals.

## Figures and Tables

**Table 1:** Physical characteristics of study participants at the time of surgery (*n* = 15) and three months post-surgery (*n* = 8).

	Surgery (*n* = 15)	Post-Surgery (*n* = 8)	*p*-Value
Age (years)	37 ± 6	35 ± 6	0.455
Sex	2 ♂, 13 ♀	1 ♂, 7 ♀	
Height (cm)	164.9 ± 6.0	166.8 ± 7.7	0.518
Body weight (kg)	132.5 ± 10.4	119.2 ± 3.5 *	0.002
BMI (kg/m^2^)	47.1 ± 6.3	40.7 ± 5.6 *	0.025
Waist circumference (cm)	131.1 ± 12.6	115.4 ± 13.8 *	0.012

* *p*-value < 0.05; BMI, body mass index.

**Table 2:** Cardiometabolic risk factors of participants at the time of surgery (*n* = 15) and three months post-surgery (*n* = 8). 1-25(OH)_2_D—1,25-dihydroxyvitamin D.

	Surgery (*n* = 15)	Post-Surgery (*n* = 8)	*p*-Value
Systolic BP (mm Hg)	130.3 ± 13.5	119.6 ± 9.8 *	0.042
Diastolic BP (mm Hg)	78.1 ± 9.1	66.7 ± 3.7 *	0.003
Heart rate (beats/min)	85.2 ± 12.9	84.5 ± 10.8	0.897
Total cholesterol (mg/dL)	185.9 ± 20.8	168.7 ± 19.3 *	0.044
Triglycerides (mg/dL)	78.8 ± 10.2	77.3 ± 16.5	0.789
Glucose (mg/dL)	87.4 ± 15.4	83.8 ± 13.3 *	0.047
Insulin (µIU/mL)	10.1 ± 2.0	9.7 ± 1.3	0.616
1-25(OH)_2_D (ng/mL)	13.8 ± 1.8	18.1 ± 2.2 *	0.0003

* *p*-value < 0.05; BP, blood pressure.
